# The Question of the Narrative in Historiography: Between the Annales School and Hayden White

**DOI:** 10.12688/openreseurope.20777.1

**Published:** 2025-08-27

**Authors:** Karlo Držaić

**Affiliations:** 1 University of Zagreb, Faculty of Humanities and Social Sciences, Zagreb, 10000, Croatia

**Keywords:** Hayden White, Annales School, narrativity, historiography, literature

## Abstract

This paper examines the contrasting approaches of the Annales School and Hayden White to some key concepts of historiography. The historical narrative is taken as central problem around which two positions, Annales’ rejection of narrative as unscientific and White’s assertion that narrative is an unavoidable framework shaping historical understanding, are constructed. Ultimately, the paper underscores the implications of this positions in regard to the question of distinction between history and literature, as well as in regard to the concepts of truth and referentiality.

In the history of twentieth-century historiography, there is probably no more influential stream of thought than that which, over several generations, gathered around the journal *Annales. Economies, sociétés, civilisations*, and is therefore mostly known as the Annales School ^1^. It can rightfully be argued that the influence of the Annales School on practices, methods, fields of study, and ideas in historiography was radical. This influence meant a departure from the then traditional conception of historiography with an emphasis on political history, rejection of the linear and causal narrative model, connection with social sciences, encouragement to explore new themes and areas such as economic and social structures or mentality, and so on. From its inception, either as an answer to the perceived crisis of history or as a negation of previous historiography, the ideas of the Annales School were accepted or appropriated throughout the twentieth century and perhaps even became dominant in what we might nominally call European *disciplinary* historiography. Lefebvre, Bloch, Braudel, Nora, Le Goff and others are without a doubt canonical in any examination of the European historiography, and today, the assertion from my first sentence about the influence of the Annales School on historiography no longer requires much explanation or argumentation. The Annales School functions as a main role model of *normal* history, of the kind of history that is most widely practiced and the kind that concerns itself far more with the past, then with the contemporary developments in the field of theory of history. ^2^ In this context, it is not necessary for this *normal* history to consistently follow the practices of the Annales school, such as a focus on the *longue durée* or non-narrative history. Instead, it can also be limited to positivist tendencies, the idea of promoting history as a firm science through interdisciplinarity, and similar approaches.

The situation is somewhat different with the other protagonist of this paper, Hayden White. Similar to the Annales School, White had a significant influence on contemporary historiography, and his work *Metahistory: The Historical Imagination in Nineteenth-century Europe*, published in 1973, is generally considered the moment of entry of the linguistic and rhetorical turn in historiography (cf. 1 on page 185). However, White's critical approach, his questioning of the basic postulates of scientific historiography, or rather, those postulates that are often considered essential for promoting the study of history to the level of real science, has earned him a place on the margins of the historiographic field. His work is often overlooked as a casual mention of an attempt to deny historiography the possibility of knowledge of history or the value of historiography, and it plays a significantly larger role in disciplines such as literary studies and philosophy, then it does in history itself. White is not alone here but occupies the same position that in historiography traditionally belongs to the philosophy of history, or theory. This is in line with the stance long ago expressed by Jakob Burckhardt, “The philosophy of history is a centaur, a contradiction in terms, for history coordinates and hence is unphilosophical, while philosophy subordinates and hence is unhistorical.” ( 3 on page 15)

The Annales School and Hayden White, due to their influence, figure in the field of historiography as symbols of opposing traditions. The Annales School represents dominant, *normal*, historiography as a scientific discipline with its rules, outcomes, and expectations, and on the other hand, Hayden White embodies the critique of such historiography, including critiques from other authors such as Ankersmith, LaCapra, Ricoeur, Hans Kellner or others. Given this, White often plays the role of a subversive factor in the context of historiography, more precisely in the filed of theory of history, one that potentially undermines the foundations of the *historian’s craft*. One of the key concepts around which the theories of history from the Annales School and those of Hayden White, as well as most other poststructuralist theorists of history and historiography, clash is the question of the role and function of narrative and narrativity. From antiquity and the first historians to the present day, narrative has served as the basic model of historiography, simply put, historians have always told stories. ^3^ However, from the first half of the twentieth century, and especially after World War II, a new tendency has emerged to distance historiography from narrative history and its *story-telling* function, moving it toward an analysis that is not event-based and through which historiography might align more closely with the social sciences. Given this, in this paper I will outline the problem of historical narrative from the perspective of the Annales School on one hand, and Hayden White on the other. Moving from this outline of their complex relationship, I will attempt to draw conclusions about the role of narrative in history and the repercussions of that role on the understanding of some key concepts in historiography, as well as it’s function if we accept either of the two perspectives.

In addition to what has already been mentioned, there are several other reasons supporting the validity of comparing the Annales School and White. A simple, but relevant reason is the temporal coincidence. Namely, just one year after White published *Metahistory: The Historical Imagination in Nineteenth-century Europe*, in 1974, Jacques Le Goff and Pierre Nora published a series of essays titled *Constructing the Past: Essays in Historical Methodology* ^4^, which can be considered a moment of gathering for the third and, arguably, last generation of the Annales School and the beginning of the New History movement. ^4^ From then until the 1990s, the Annales School and White operated in parallel, starting from mostly opposing ideas but almost without any direct debate. ^5^ The third reason I chose these starting points is that White's theory and the Annales historians' idea of history clash precisely on the levels of basic concepts of historiography, such as truth, referentiality and especially narrative.

However, it should be noted that this comparison is problematic on several levels. Firstly, it requires a significant reduction both of White, who slightly changed his positions over decades of work since the publication of *Metahistory*, and of the historians classified within the Annales School, who are numerous and operated throughout the second half of the twentieth century. Moreover, the historians gathered around the *Annales* journal mostly claimed that they did not belong to any common school but were characterized by a shared spirit of openness to the use of new methods and approaches in historical research. ^6^ However, it cannot be denied that generations of these historians are defined by common features and concepts, as well as by an affiliation with certain institutions, including the journal itself, all of which all characteristics of a school of thought. Another issue with the Annales School lies in what we might call omission of theory, or sometimes even aversion to theory, in accordance with the traditional relationship of historiography to the philosophy of history. Therefore, the Annales School never developed a unifying and concrete philosophy of history. An example of this omission of theory by the Annales historians is the famous book *The Historian's Craft* ( 9) ^7^, written by Marc Bloch. Although mostly writing without having sources and literature at hand, Bloch deals more with concreate historical examples and analogies than with contemplating the theory of historiography. However, even with this, his approach shows the school's broader inclination towards understanding history through practice and experience rather than through abstract theory. This focus meant that for the Annales historians the theory of history is mostly exhausted on the level of methodology. Nonetheless, as mentioned, the historical writings of members of the Annales School share certain common practices and concepts that reflect their understanding of theoretical principles in historiography.

Following this, in some aspects the works of members of the Annales School show significant similarities. For example, we can highlight Bloch's *Feudal Society* ^10^, Braudel's *The Mediterranean and the Mediterranean World in the Age of Philip II* ^11, 12^, his *Civilisation and Capitalism* ^13^, or Le Roy Ladurie's *Peasants of Languedoc* ^14^, which, like many other capital works of the Annales historians, are united by the absence of institutions or (great) people acting as protagonists of the historical narrative, by focus on *long durée* phenomena, and by distancing from *l'histoire événementielle*. In this way, the Annales historians seek to move away from a linear and causal conception of history, which is necessary teleological. ^8^ A lasting reason for this must be sought in a different understanding of historical time, which is no longer determined by a relatively short sequence of events, nor is it uniform. Braudel in *The Mediterranean and the Mediterranean World in the Age of Philip II*, despite the title, gives only a secondary role to the narrative of events in the Mediterranean during the time of Philip II, focusing much more on the structures of the long duration in geographical space and transgenerational phenomena of society and culture. Simmillary Le Goff in the essay “Church time and merchant time in the Middle Ages” ^17^ further highlights the multiplicity of historical time, and thus the multiplicity of history. Shifting the research focus from the course of events to phenomena allowed history to be defined not exclusively through change but also to define and investigate the almost static nature, or even *immobility*, of a particular phenomenon or individual event through historical categories ( 15 on pages 396–397). In the article “History and the Social Sciences: The Long Duration”, in which Braudel introduces the concept of the *longue durée* as a point of connection between the social sciences and history, he discusses historical narrative only at the levels of events and conjunctures, that is, on the levels of short and medium duration ( 18 on pages 5–7). On the other hand, he emphasizes that the acceptance of long-duration structures “signifies a change in style and attitude” and encourages the historian to “slow down time” almost to the point of stasis ( 18 on page 7). Since time or movement is a fundamental characteristic of the conventional concept of narrative, a history that is almost static, for Braudel and the Annales School, is a history that does not need narrative, one in which narrative seems impossible. Instead of narrative, Braudel and some Annales historians use the concept of a historical *tableau.* ^9^ Inspired by Jules Michelet and his *Tableau de la France* (1875), the historical *tableau* is a static textual representation of that history which is imagined as a picture or even a photograph. All this necessarily meant abandoning linear historical time, but also the old conception according to which the narrative, despite similarities to literature but due to the methodology of history, functions as a mimesis, or at least a valid representation, of past reality.

Another reason for rejecting the historical narrative is the attempt to bring historiography closer to the social sciences and move it away from literature, or to affirm its status as a “real” science. From the beginning, the Annales School emphasized the need for interdisciplinarity with the social sciences, but this intensified from the late 1960s, in line with a general trend in historiography that ultimately led to quantitative history and cliometrics. ^10^ Precisely the effort to address criticisms about the speculative nature of historiography and to make it scientific according to the standards of the social sciences is one of the main and lasting characteristics of the Annales School, from Bloch to the late twentieth century. Thus, Le Roy Ladurie points out that, by the mid-twentieth century, strong voices from the social sciences were calling for the rejection of historiography as a *soft* science that lacks the rigorous methods of scientific research, yet that such a stance stems either from ignorance or malice, because “since Bloch, Braudel, and Labrousse, history had also effected its scientific mutation.” ( 20 on pages 135–136) Le Roy Ladurie continues by asserting that history “had surprised the social sciences at the swimming hole and made off with their clothes, and the victims had not even noticed their nakedness.” With this, he argues not only that history is no less scientific than the social sciences but also that, after adopting the methods of the social sciences, historiography alone can adequately speak about the human past. ( 20 on pages 135–136) In a similar vein, Furet emphasizes that historiography, in its “renewed form”, which abandons the claims of historicism and with them the ideas of linear historical progress, does not merely borrow methods from the social sciences but instead blurs the boundaries between these two fields and even harbours hegemonic ambitions over the social sciences, “as if history alone, armed with the partial forms of knowledge developed by neighbouring disciplines, were destined to present a unified vision of man under the name of ‘total history’.” ( 15 on pages 393–394)

Similarly, and in contrast to the traditional understanding of historiography, Furet, in his 1971 essay “Quantitative History” opposes event history because it is “made up of a mere narrative of certain selected 'events' along the time axis and based on the idea that these events are unique and cannot be set out statistically, and that the unique is the material par excellence of history.” ( 21 on page 160) He later elaborates on this position by stating that “the traditional historical explanation obeys the logic of the narrative,” and is governed by “this implicit logic, which privileges the period over the object, and selects events based on their place in a narrative, defined by a beginning and an end.” ( 15 on pages 396–397) Accordingly, the basis of the general critique by the Annales School towards narrative and narrative history stems from the notion that it is a form of writing history that, due to its similarity to and origin in literature, is insufficiently scientific and can only present a simplified, superficial, and seemingly absolute version of past reality, typically through the privileged position of political history. Along these lines, reflecting on the past and future of the Annales School, Furet concludes on the function and place of narrative in historiography, saying that narrative: “endows scholarship and archival research with the charm and even the pleasure of a novel. Because it is built on a succession of concrete and unique facts, it calls on the historian’s evocative powers more than on his specifically intellectual ability, on his art more than on his mind, on his sensitivity more than on his intelligence.” ( 15 on page 406)

Hayden White rarely explicitly addresses these ideas of the Annales historians, but in contrast to them, White emphasizes that narrative is not just a form but that it also has the function of narrativization, which acts as an apparatus for creating meaning. This is done through the imposition of narrative as a discursive form on events and figures in history by means that are necessary poetic in nature. In his seminal work *Metahistory*, White puts forth a thesis that history is necessarily narratively prefigured through the use of four tropes, lines of argumentation and ideological implications ^22^. White emphasizes that it is the historian who creates the field of their interest at the very level of conceptualizing the past and that they necessarily create the figures and relationships that define this field.

White provides the example of medieval annals, specifically the records of the Saint Gall monastery, where, over a few centuries, one or two unrelated events were recorded each year without placing them in any hierarchy. Events such as floods, deaths, the arrival of the Saracens, years of good harvest, or the crowning of kings were recorded as facts without any moral-political structure ( 23 on pages 6–18). Based on such examples of medieval annals, White argues that it is not enough for events from history to be chronologically arranged for us to consider them a historical account. It is also not sufficient for them to be connected in some form of mutual relationship or plot, but also necessary is the moment of narrativization, which endows history with meaning. According to White, this essentially means disciplining history through the moral-political context of the historian, implying that the narrativization of events carries an imprint of content that is foreign to history itself. ^11^ Hayden White emphasizes that, what he calls, *real events*, in themselves, do not carry intrinsic narratives or emotional tones such as tragedy, comedy, or farce. Instead, meaning is assigned to events through the process of narrativization, by selecting a certain type of story and imposing it on those events. ( 24 on pages 83–84) In other words, the same set of events can be interpreted and told in different ways, depending on the narrative framework, or story type, the historian decides to use. Or, as White puts it:

The production of meaning, in this case, can be regarded as a performance, because any given set of real events can be emplotted in a number of ways, can bear the weight of being told as any number of different kinds of stories. Since no given set or sequence of real events is intrinsically 'tragic,' 'comic,' or 'farcical,' but can be constructed as such only by the imposition of the structure of a given story-type on the events, it is the choice of the story-type and its imposition upon the events which endow them with meaning. ( 25 on page 20)

In other words, White's concept of narrative is radically different from that of the historians of the Annales School. For White, narrative as a form also carries a certain function, or *content*, which influences what is narrated. Narrative is actually an autonomous function that is not determined by the subject matter the historian addresses. Likewise, the historical narrative is a complex whole that cannot be reduced to its basic elements, to *real events*.

Following this line of reasoning, White, in his debate with the historian Georg Iggers, develops the argument that the narrative storytelling of historians, whether consciously or unconsciously, is already determined by the ending they expect or the method they have decided to apply for analysis before writing ( 26 on pages 393–395). In this way, the historian shapes the entire narrative and gives it an internal consistency that leads towards a outcome or at least allows the application of a pre-chosen method. This again means that history, from the aspect of its writing, is inevitably teleological, even if the text itself on the surface does not have such characteristics. In this sense, White's critique of narrative in history is at least twofold. On one hand, it relates to the problem of surplus that necessarily arises in giving moral and political meaning to historical events, and this surplus already occurs at the point of selection of events. On the other hand, the historical narrative represents a closed whole that the historian builds based on the logic inherent in a narrative style. This means that we cannot formally evaluate the truthfulness of a historiographical narrative except in the aspect related to chronology. ^12^ Or to use an example that White often employs, the truthfulness of Marx's narrative about the events of 1848 in France as a farcical reenactment of the tragedy of 1789, can be assessed according to the criteria of the accuracy of the event chronology, that is, whether they occurred and how, but we have no other criteria, logical or empirical, to assess the truthfulness of the narrative itself, except for the logic by which the historian himself created the narrative, and by that measure, the narrative will certainly be true. ( 26 on page 394)

Therefore, if we view narrative as a form of writing history, for members of the Annales School, it is too closely related to literature and literary discourse, in difference from which Western historiography is constituted upon, and it does not allow the conveyance of their conception of history. They believe that conventional historical narratives, which often rely on linear chronology and the dramatization of events, cannot fully capture the complexity of historical processes or the deeper causes behind historical changes, nor can they affirm historiography as a “true” science. ^13^ But the Annales historians themselves often reduce the historical narrative to a tradition of historiography. That is, they largely identify the narrative with a simple event-based political history which, although positivist and claiming to be scientific, was mostly written under the influence of national ideologies. When not reduced to this level of historiographical tradition, the narrative is viewed by the Annales school as merely a style of writing, an atavism, like something long ago borrowed from mythology, and now inadequate when history evolved into science. In opposition to that, Hayden White, in his work, approaches narrative as a key element in the construction of historical knowledge. According to White, the narrative is not just a neutral or transparent medium for conveying *facts* about the past; instead, it is a means through which history is interpreted, organized, and presented. Narrative, in White's understanding, involves processes of selection, emphasis, and connection of historical events in ways that produce meaning and coherence. The narrative is fundamentally an interpretive act that makes the past understandable through contemporary categories, and by which the historian participates in the creation of the meaning of history, making historical writing a subjective and creative act.

Therefore, Annales historians and Hayden White approach the problem of historical narrative on two different levels. For the Annales School, narrative is primarily a style of presenting knowledge about history which, at least when dealing with shorter periods, can be viewed as a narrative but also as an almost static *tableau*. For White, however, narrative is the fundamental model for understanding history and organizing knowledge about it. Thus, while Annales historians believe that narrative must be abandoned, White cannot even make such a suggestion, as narrative is unavoidable. Although these two approaches differ and conflict greatly, one aspect of a common critique of narrative in historiography can nonetheless be discerned. That the narrative is not truly representational or even referential with respect to the past it deals with. It does not and cannot correspond to the past as it happened. This understanding of narrative leads to a critical reassessment of the referentiality of narrative historiography where each historiographical text not only relates to the past but also actively shapes it through the narrative and analytical choices of the historian. This problem of referentiality opens up questions about the scope of historiography, the possibility of it representing the real past, or conveying the truth about the past. As mentioned, for the Annales and like-minded historians, the path to solving this problem lies in rejecting the classical narrative, applying scientific methods, but also in attempting to write a total history of society by dealing with previously neglected topics. That is, for them, historiographical discourse differs from literature in two ways: first, because the subject matter it deals with is reality, not fiction, and second, because it applies the methodology of science. White's approach is nowadays more interesting. He postulates the idea that there is no such essential difference between historiography and literature. The historian uses models and means of literature to convert events into a comprehensible whole, but even before that, he configures his own understanding of the past as a discursive field in which the subject matter of reality appears as fictional structures and figures. Therefore, he uses the concept of *truth-claiming intention* to differentiate history from literature. As Nancy Partner summarises, for White “history is the kind of complex prose that encrypts truth-claiming intention and reaches for meaning by deploying the formal resources of language to create intelligible and logically connected emplotments of events.” ( 27 on page 169)

However, in this context, the concept of *truth-claiming intention* is inadequate, or at least problematic. The assertion of a text’s truthfulness, its representational or even referential nature in relation to presumed historical reality is by no means exclusive to the domain of history as opposed to literature. The most obvious example of this is certainly realist literature, which Fredric Jameson defines as “a hybrid concept, in which an epistemological claim (for knowledge or truth) masquerades as an aesthetic ideal.” ( 28 on pages 3–7) This epistemological claim to truth in realism, or in other literary movements, is fundamentally no different from that in history. ^14^ Of course, while it is essential for a historical text to contain an assertion that it is truthful and referential to an actual event and period, literature may claim to be *truer than truth* because it does not need a real event to be referential to a given period, place, or other phenomenon. For describing non-narrative historiography, like that of the Annales historians, we might employ a slightly modified version of Jameson’s definition of realism. ^15^ Non-narrative historiography is a hybrid model in which the aesthetic ideal masquerades as an epistemological claim and method. The inability to fully replace literary character with the concept of photographic representation defines the hybridity of this model, which ultimately aims to substitutes aesthetics with epistemology. However, just as writing without an aesthetic ideal merely means writing with a different aesthetic ideal, so too does the attempt to create a history without narrative, even when this history is (almost) static, result only in the creation of a different narrative or, at the very least, leaves a larger role to the reader in forming their own narrative. After all, it is nearly impossible to read some of the great historians of the Annales School without noticing their exceptional literary skill and developed aesthetic. Perhaps the best example is *The Mediterranean*, in which Braudel, avoiding historical narrative, writes an extraordinary epic in which the Mediterranean itself emerges as the subject.

If we choose to follow White’s claim that the truthfulness of a historical narrative can be determined only in relation to chronology, or to the particular, we lose any possibility of using an external and objective measure to determine whether history is more truthful than literature, regardless of literature's self-acknowledged fictional nature. Such an understanding of representation and truth departs from the positivist and empiricist ideas of the Annales School and the majority of mainstream historiography, designating the value of truth only to the realm of metaphor and allegory, a realm where literature can also rightfully claim its truthfulness. Given this, the approach to historical narrative is not a point at which historiography can draw a clear line of distinction from literature and prove its belonging to the firm sciences, not even in the case of the Annales School’s history without narrative. However, the conclusion of White’s general approach to history is not necessary to deny the potential of history to speak truth about the past. On the contrary, his critique opens new space for historiography but also calls for its redefinition, the abandonment of traditional notions of truth, and a shift in the field within which historiography functions. ^16^

By distinguishing between truth and its establishment, as well as between history and literature, Chris Lorenz emphasizes that the “intersubjective character of the rules of ‘doing history’ in contrast to ‘doing literature’” is what “constitutes history’s distinguishing hallmark as an empirical discipline.” ( 29 on page 326) ^17^ For him, the intersubjective character of the rules of history is essentially the process of exposition, argumentation, and questioning, namely, a process of validation according to various procedures and criteria applied by historians, including the assumption that the historian has first conducted research ( 29 on pages 326–327). However, Lorenz’s distinction is not entirely adequate. What he terms “doing literature” in no way implies that a writer does not undertake serious and rigorous research on his subject, and such research may align with the methods of history. Moreover, it is impossible to deny the intersubjectivity of literature, especially in certain genres. The most obvious example is likely the historical novel, in which public debate about historical accuracy, including chronological and factual accuracy, is entirely unavoidable. Nevertheless, the intersubjectivity of history, not only as a feature or characteristic but as the very definition and site of history-making, remains crucial. In this context, I refer to Foucault’s concept of a *regimes of truth*. In the interview *Truth and Power*, first published in 1977, Foucault argues that every society possesses “its regime of truth, its ‘general politics’ of truth; that is, the types of discourse which it accepts and makes function as true.” ( 32 on page 131) What Lorenz describes as the process of validation in history according to intersubjective rules, that is, the procedures and criteria of historiography, is elevated in Foucault’s framework to a broader social and historical context. This includes the “mechanisms and instances which enable one to distinguish true and false statements, the means by which each is sanctioned; the techniques and procedures accorded value in the acquisition of the truth; the status of those who are charged with saying what counts as true.” ( 32 on page 131)

Following Foucault’s concept of the regimes of truth, historiography should be defined through the relationship between the text and the society in which the question of truth is privileged as the dominant factor determining the nature of that relationship. Different societies, or different discourses, apply their own mechanisms, or power relations, to determine which statements, or, on a higher-level which narratives, are true and which are false. Alternatively, as Jouni-Matti Kuukkanen suggests in his shift from the framework of truth-functionality “to the framework of rational warrants and rational appropriateness,” the mechanisms define which statements and thesis are appropriate, fitting or unfitting, useful or useless, and so on. ( 31 on page 234) ^18^ By defining the relationship between the text and society (its audience) as the locus where historiography is constituted, both historiography and the text itself gain epistemic authority only in a retroactive relationship. That is, only when all the elements that traditionally constitute historiography, such as research and methodology, are recognized as science and as historiography that, to some extent at least, corresponds with the past. In this context, the key roles are not played by the source material, the methods used in the research itself, or even the modes of narrativization, but rather by the mechanisms of a given regime of truth. However, the assumption that certain source materials were used, that the research was conducted according to certain methodologically, and that the process of narrativization occurred are prerequisites for a text to be recognised as historiography, at least within the context of modern Western history. Only here is it possible to establish a distinction between history and literature that aligns with the narrativist critique of history. This distinction no longer stems from the intrinsic character or rules of historiography itself, such as the Annales School’s attempt to reject narrative, but is constituted as a specific construct. Historiography is accepted as a discourse with the exclusive capacity to speak the truth about history, based on two separations, the separation of the present from the past and the separation of the historian from history.

Defined in this way, as a discourse fixed through the relationship between text and society and with regard to regimes of truth, historiography can break free from certain self-imposed epistemic and methodological limitations. Following White's conclusion about the political potential of the meaninglessness of history, we can infer that the meaninglessness of historiography can goad historians to construct histories with a meaning for which they alone are fully responsible. ( 30 on page 72) That is, to construct truly new histories and new historiographies. Or the evocate Marx oppening words of his classic *The Eighteenth Brumaire of Louis Bonaparte*, “The tradiotion of all past generations weighs like a nightmare on the brains of the living.” Furthermore, such a definition actually re-establishes the relevance of the theories and practices of the Annales School, including its attempt to write history without narrative. Moreover, in the context of modern Western historiography and contemporary Western society, which, at least as much as in the mid-twentieth century, grants a privileged position to the firm sciences as being more capable of speaking truth than the humanities, the Annales School can occupy a privileged position precisely because it masquerades as a firm science. In this way, the consequence of White's critique of historical narrative and history in general, as well as the critiques of other theorists with of simmilar orientation, is not necessarily the disintegration of history as a discipline but its liberation from self-disciplining. In such a historiography, both the non-narrative narrative of history promoted by the Annales School and White's tropological analysis could potentially be equally legitimate approaches to history.

## Ethics and consent

Ethical approval and consent were not required.

## Data Availability

No data associated with this article.
