# The Question of the Narrative in Historiography: Between the Annales School and Hayden White

**DOI:** 10.12688/openreseurope.20777.2

**Published:** 2026-04-02

**Authors:** Karlo Držaić

**Affiliations:** 1 University of Zagreb, Faculty of Humanities and Social Sciences, Zagreb, 10000, Croatia

**Keywords:** Hayden White, Annales School, narrativity, historiography, literature

## Abstract

This paper examines the contrasting approaches of the Annales School and Hayden White to some key concepts of historiography. The historical narrative is taken as central problem around which two positions, Annales’ rejection of narrative as unscientific and White’s assertion that narrative is an unavoidable framework shaping historical understanding, are constructed. Ultimately, the paper underscores the implications of this positions in regard to the question of distinction between history and literature, as well as in regard to the concepts of truth and referentiality.

In the history of twentieth-century historiography, there is probably no more influential stream of thought than that which, over several generations, gathered around the journal *Annales. Economies, sociétés, civilisations*, and is therefore mostly known as the Annales School [ 1]. It can rightfully be argued that the influence of the Annales School on practices, methods, fields of study, and ideas in historiography was radical. This influence meant a departure from the then traditional conception of historiography with an emphasis on political history, rejection of the linear and causal narrative model, connection with social sciences, encouragement to explore new themes and areas such as economic and social structures or mentality, and so on. From its inception, either as an answer to the perceived crisis of history or as a negation of previous historiography, the ideas of the Annales School were accepted or appropriated throughout the twentieth century and perhaps even became dominant in what we might nominally call European *disciplinary* historiography. Lefebvre, Bloch, Braudel, Nora, Le Goff and others are without a doubt canonical in any examination of the European historiography, and today, the assertion from my first sentence about the influence of the Annales School on historiography no longer requires much explanation or argumentation. The Annales School functions as a main role model of *normal* history, of the kind of history that is most widely practiced and the kind that concerns itself far more with the past, then with the contemporary developments in the field of theory of history [ 2]. In this context, it is not necessary for this *normal* history to consistently follow the practices of the Annales school, such as a focus on the *longue durée* or non-narrative history. Instead, it can also be limited to positivist tendencies, the idea of promoting history as a firm science through interdisciplinarity, and similar approaches.

The situation is somewhat different with the other protagonist of this paper, Hayden White. Similar to the Annales School, White had a significant influence on contemporary historiography, and his work *Metahistory: The Historical Imagination in Nineteenth-century Europe*, published in 1973, is generally considered the moment of entry of the linguistic and rhetorical turn in historiography (cf. 1 on page 185). However, White’s critical approach, his questioning of the basic postulates of scientific historiography, or rather, those postulates that are often considered essential for promoting the study of history to the level of real science, has earned him a place on the margins of the historiographic field. His work is often overlooked as a casual mention of an attempt to deny historiography the possibility of knowledge of history or the value of historiography, and it plays a significantly larger role in disciplines such as literary studies and philosophy, then it does in history itself. White is not alone here but occupies the same position that in historiography traditionally belongs to the philosophy of history, or theory. This is in line with the stance long ago expressed by Jakob Burckhardt, “The philosophy of history is a centaur, a contradiction in terms, for history coordinates and hence is unphilosophical, while philosophy subordinates and hence is unhistorical.” ( 3 on page 15).

The Annales School and Hayden White, due to their influence, figure in the field of historiography as symbols of opposing traditions. The Annales School represents dominant, *normal*, historiography as a scientific discipline with its rules, outcomes, and expectations, and on the other hand, Hayden White embodies the critique of such historiography, including critiques from other authors such as Ankersmith, LaCapra, Ricoeur, Hans Kellner or others. Given this, White often plays the role of a subversive factor in the context of historiography, more precisely in the field of theory of history, one that potentially undermines the foundations of the *historian’s craft.* One of the key concepts around which the theories of history from the Annales School and those of Hayden White, as well as most other poststructuralist theorists of history and historiography, clash is the question of the role and function of narrative and narrativity. From antiquity and the first historians to the present day, narrative has served as the basic model of historiography, simply put, historians have always told stories [ 3]. However, from the first half of the twentieth century, and especially after World War II, a new tendency has emerged to distance historiography from narrative history and its *story-telling* function, moving it toward an analysis that is not event-based and through which historiography might align more closely with the social sciences. The question of narrative has been central to historical theory at least since the 1970s, and recent scholarship has substantially refined the terms of debate. For example, Herman Paul’s intellectual biography of White ( 4) offers a detailed account of the development and shifts in White’s position on narrative across his career while Kalle Pihlainen has further elaborated the implications of White’s narrativism for the practice of contemporary historiography ( 5). The present paper engages with this body of scholarship in order to clarify what is at stake in the opposition between the Annales School and White, and to argue that, despite their divergence, both traditions share a fundamentally critical stance toward conventional historical narrative. While scholars such as Frank Ankersmit, Keith Jenkins and Chris Lorenz have each addressed aspects of this problematic, Ankersmit by developing a narrativist philosophy of historical representation, Jenkins by radicalising the constructivist implications of White’s critique, and Lorenz by defending the epistemological distinctiveness of historiography against narrativist scepticism, the specific question of what unites and divides the Annales School and White at the level of their epistemological presuppositions about narrative has not been systematically examined. This paper aims to address that gap. Given this, in this paper I will outline the problem of historical narrative from the perspective of the Annales School on one hand, and Hayden White on the other. Moving from this outline of their complex relationship, I will attempt to draw conclusions about the role of narrative in history and the repercussions of that role on the understanding of some key concepts in historiography, as well as it’s function if we accept either of the two perspectives.

Methodologically, this paper employs conceptual comparison as its primary analytical approach. The comparison between the Annales School and White is not conceived as a historical survey of influence or reception, but as a philosophical analysis of the underlying epistemological commitments that structure each tradition’s engagement with narrative. This entails a close reading of key theoretical texts, principally White’s Metahistory and his later essays, alongside programmatic and reflective writings by Braudel, Furet, and Le Roy Ladurie with the aim of identifying the criteria by which each tradition defines, evaluates, and either rejects or problematises historical narrative. The selection of sources is guided by their theoretical explicitness. Most of the studied texts are those in which the authors themselves reflect on the status of narrative in historical writing, rather than simply practicing a particular mode of historiography. This approach has its limitations. It necessarily brackets the question of how these theoretical commitments manifest in the full range of historical works produced within each tradition and it risks overstating the coherence of positions that were in practice more varied and contested. These limitations are acknowledged where relevant in the discussion.

In addition to what has previously been mentioned, there are several other reasons supporting the validity of comparing the Annales School and White. A simple, but relevant reason is the temporal coincidence. Namely, just one year after White published *Metahistory: The Historical Imagination in Nineteenth-century Europe*, in 1974, Jacques Le Goff and Pierre Nora published a series of essays titled *Constructing the Past: Essays in Historical Methodology* ( 6), which can be considered a moment of gathering for the third and, arguably, last generation of the Annales School and the beginning of the New History movement [ 4]. From then until the 1990s, the Annales School and White operated in parallel, starting from mostly opposing ideas but almost without any direct debate [ 5]. The third reason I chose these starting points is that White’s theory and the Annales historians’ idea of history clash precisely on the levels of basic concepts of historiography, such as truth, referentiality and especially narrative.

However, it should be noted that this comparison is problematic on several levels. Firstly, it requires a significant reduction both of White, who slightly changed his positions over decades of work since the publication of *Metahistory*, and of the historians classified within the Annales School, who are numerous and operated throughout the second half of the twentieth century. Moreover, the historians gathered around the *Annales* journal mostly claimed that they did not belong to any common school but were characterized by a shared spirit of openness to the use of new methods and approaches in historical research [ 6]. However, it cannot be denied that generations of these historians are defined by common features and concepts, as well as by an affiliation with certain institutions, including the journal itself, all of which all characteristics of a school of thought. Another issue with the Annales School lies in what we might call omission of theory, or sometimes even aversion to theory, in accordance with the traditional relationship of historiography to the philosophy of history. Therefore, the Annales School never developed a unifying and concrete philosophy of history. An example of this omission of theory by the Annales historians is the famous book *The Historian’s Craft* ^11^ [ 7], written by Marc Bloch. Although mostly writing without having sources and literature at hand, Bloch deals more with concreate historical examples and analogies than with contemplating the theory of historiography. However, even with this, his approach shows the school’s broader inclination towards understanding history through practice and experience rather than through abstract theory. This focus meant that for the Annales historians the theory of history is mostly exhausted on the level of methodology. Nonetheless, as mentioned, the historical writings of members of the Annales School share certain common practices and concepts that reflect their understanding of theoretical principles in historiography.

Following this, in some aspects the works of members of the Annales School show significant similarities. For example, we can highlight Bloch’s *Feudal Society*, ^12^ Braudel’s *The Mediterranean and the Mediterranean World in the Age of Philip II*, ^13^^,^^14^ his *Civilisation and Capitalism*, ^15^ or Le Roy Ladurie’s *Peasants of Languedoc*, ^16^ which, like many other capital works of the Annales historians, are united by the absence of institutions or (great) people acting as protagonists of the historical narrative, by focus on *long durée* phenomena, and by distancing from *l’histoire événementielle.* In this way, the Annales historians seek to move away from a linear and causal conception of history, which is necessary teleological [ 8]. A lasting reason for this must be sought in a different understanding of historical time, which is no longer determined by a relatively short sequence of events, nor is it uniform. Braudel in *The Mediterranean and the Mediterranean World in the Age of Philip II*, despite the title, gives only a secondary role to the narrative of events in the Mediterranean during the time of Philip II, focusing much more on the structures of the long duration in geographical space and transgenerational phenomena of society and culture [ 9]. Simmillary Le Goff in the essay “Church time and merchant time in the Middle Ages” ^20^ further highlights the multiplicity of historical time, and thus the multiplicity of history. A particularly instructive case is Le Goff’s concept of the anti-biography, articulated most clearly in his biography of Saint Louis ( 21). Le Goff frames his project as a rejection of traditional biographical narrative, which he associates with a focus on individual psychology, linear chronology, and the construction of a unified character. In its place, he proposes a form of historical writing centred on the social, cultural, and theological structures that shaped the figure of Saint Louis, structures that cannot be captured by conventional narrative but require the kind of structural and *longue durée* analysis characteristic of the Annales School. However, Le Goff’s anti-biography is not simply the absence of narrative, it retains a form of organization around a central figure and a loosely chronological frame, even as it systematically subverts the narrative conventions of classical biography. Such shifting of the research focus from the course of events to phenomena allowed history to be defined not exclusively through change but also to define and investigate the almost static nature, or even *immobility*, of a particular phenomenon or individual event through historical categories ( 17 on pages 396–397). In the article “History and the Social Sciences: The Long Duration”, in which Braudel introduces the concept of the *longue durée* as a point of connection between the social sciences and history, he discusses historical narrative only at the levels of events and conjunctures, that is, on the levels of short and medium duration ( 22 on pages 5–7). On the other hand, he emphasizes that the acceptance of long-duration structures “signifies a change in style and attitude” and encourages the historian to “slow down time” almost to the point of stasis ( 22 on page 7). Since time or movement is a fundamental characteristic of the conventional concept of narrative, a history that is almost static, for Braudel and the Annales School, is a history that does not need narrative, one in which narrative seems impossible. Instead of narrative, Braudel and some Annales historians use the concept of a historical *tableau* [ 10]. Inspired by Jules Michelet and his *Tableau de la France* (1875), the historical *tableau* is a static textual representation of that history which is imagined as a picture or even a photograph. All this necessarily meant abandoning linear historical time, but also the old conception according to which the narrative, despite similarities to literature but due to the methodology of history, functions as a mimesis, or at least a valid representation, of past reality.

Another reason for rejecting the historical narrative is the attempt to bring historiography closer to the social sciences and move it away from literature, or to affirm its status as a “real” science. From the beginning, the Annales School emphasized the need for interdisciplinarity with the social sciences, but this intensified from the late 1960s, in line with a general trend in historiography that ultimately led to quantitative history and cliometrics [ 11]. Precisely the effort to address criticisms about the speculative nature of historiography and to make it scientific according to the standards of the social sciences is one of the main and lasting characteristics of the Annales School, from Bloch to the late twentieth century. Thus, Le Roy Ladurie points out that, by the mid-twentieth century, strong voices from the social sciences were calling for the rejection of historiography as a *soft* science that lacks the rigorous methods of scientific research, yet that such a stance stems either from ignorance or malice, because “since Bloch, Braudel, and Labrousse, history had also effected its scientific mutation.” ( 24 on pages 135–136). Le Roy Ladurie continues by asserting that history “had surprised the social sciences at the swimming hole and made off with their clothes, and the victims had not even noticed their nakedness.” With this, he argues not only that history is no less scientific than the social sciences but also that, after adopting the methods of the social sciences, historiography alone can adequately speak about the human past. ( 24 on pages 135–136) In a similar vein, Furet emphasizes that historiography, in its “renewed form”, which abandons the claims of historicism and with them the ideas of linear historical progress, does not merely borrow methods from the social sciences but instead blurs the boundaries between these two fields and even harbours hegemonic ambitions over the social sciences, “as if history alone, armed with the partial forms of knowledge developed by neighbouring disciplines, were destined to present a unified vision of man under the name of ‘total history’.” ( 17 on pages 393–394).

Similarly, and in contrast to the traditional understanding of historiography, Furet, in his 1971 essay “Quantitative History” opposes event history because it is “made up of a mere narrative of certain selected ‘events’ along the time axis and based on the idea that these events are unique and cannot be set out statistically, and that the unique is the material par excellence of history.” ( 25 on page 160) He later elaborates on this position by stating that “the traditional historical explanation obeys the logic of the narrative,” and is governed by “this implicit logic, which privileges the period over the object, and selects events based on their place in a narrative, defined by a beginning and an end.” ( 17 on pages 396–397) Accordingly, the basis of the general critique by the Annales School towards narrative and narrative history stems from the notion that it is a form of writing history that, due to its similarity to and origin in literature, is insufficiently scientific and can only present a simplified, superficial, and seemingly absolute version of past reality, typically through the privileged position of political history. Along these lines, reflecting on the past and future of the Annales School, Furet concludes on the function and place of narrative in historiography, saying that narrative: “endows scholarship and archival research with the charm and even the pleasure of a novel. Because it is built on a succession of concrete and unique facts, it calls on the historian’s evocative powers more than on his specifically intellectual ability, on his art more than on his mind, on his sensitivity more than on his intelligence.” ( 17 on page 406).

Hayden White rarely explicitly addresses these ideas of the Annales historians, but in contrast to them, White emphasizes that narrative is not just a form but that it also has the function of narrativization, which acts as an apparatus for creating meaning. This is done through the imposition of narrative as a discursive form on events and figures in history by means that are necessary poetic in nature. In his seminal work *Metahistory*, White puts forth a thesis that history is necessarily narratively prefigured through the use of four tropes, lines of argumentation and ideological implications. ^26^ White emphasizes that it is the historian who creates the field of their interest at the very level of conceptualizing the past and that they necessarily create the figures and relationships that define this field [ 12].

White provides the example of medieval annals, specifically the records of the Saint Gall monastery, where, over a few centuries, one or two unrelated events were recorded each year without placing them in any hierarchy. Events such as floods, deaths, the arrival of the Saracens, years of good harvest, or the crowning of kings were recorded as facts without any moral-political structure ( 27 on pages 6–18). Based on such examples of medieval annals, White argues that it is not enough for events from history to be chronologically arranged for us to consider them a historical account. It is also not sufficient for them to be connected in some form of mutual relationship or plot, but also necessary is the moment of narrativization, which endows history with meaning. According to White, this essentially means disciplining history through the moral-political context of the historian, implying that the narrativization of events carries an imprint of content that is foreign to history itself [ 13]. Hayden White emphasizes that, what he calls, *real events*, in themselves, do not carry intrinsic narratives or emotional tones such as tragedy, comedy, or farce. Instead, meaning is assigned to events through the process of narrativization, by selecting a certain type of story and imposing it on those events. ( 28 on pages 83–84) In other words, the same set of events can be interpreted and told in different ways, depending on the narrative framework, or story type, the historian decides to use. Or, as White puts it:

The production of meaning, in this case, can be regarded as a performance, because any given set of real events can be emplotted in a number of ways, can bear the weight of being told as any number of different kinds of stories. Since no given set or sequence of real events is intrinsically ‘tragic,’ ‘comic,’ or ‘farcical,’ but can be constructed as such only by the imposition of the structure of a given story-type on the events, it is the choice of the story-type and its imposition upon the events which endow them with meaning. ( 29 on page 20)

In other words, White’s concept of narrative is radically different from that of the historians of the Annales School. For White, narrative as a form also carries a certain function, or *content*, which influences what is narrated. Narrative is actually an autonomous function that is not determined by the subject matter the historian addresses. Likewise, the historical narrative is a complex whole that cannot be reduced to its basic elements, to *real events.*

Following this line of reasoning, White, in his debate with the historian Georg Iggers, develops the argument that the narrative storytelling of historians, whether consciously or unconsciously, is already determined by the ending they expect or the method they have decided to apply for analysis before writing ( 30 on pages 393–395). In this way, the historian shapes the entire narrative and gives it an internal consistency that leads towards a outcome or at least allows the application of a pre-chosen method. This again means that history, from the aspect of its writing, is inevitably teleological, even if the text itself on the surface does not have such characteristics. In this sense, White’s critique of narrative in history is at least twofold. On one hand, it relates to the problem of surplus that necessarily arises in giving moral and political meaning to historical events, and this surplus already occurs at the point of selection of events. On the other hand, the historical narrative represents a closed whole that the historian builds based on the logic inherent in a narrative style. This means that we cannot formally evaluate the truthfulness of a historiographical narrative except in the aspect related to chronology [ 14]. Or to use an example that White often employs, the truthfulness of Marx’s narrative about the events of 1848 in France as a farcical reenactment of the tragedy of 1789, can be assessed according to the criteria of the accuracy of the event chronology, that is, whether they occurred and how, but we have no other criteria, logical or empirical, to assess the truthfulness of the narrative itself, except for the logic by which the historian himself created the narrative, and by that measure, the narrative will certainly be true. ( 30 on page 394).

Therefore, if we view narrative as a form of writing history, for members of the Annales School, it is too closely related to literature and literary discourse, in difference from which Western historiography is constituted upon, and it does not allow the conveyance of their conception of history. They believe that conventional historical narratives, which often rely on linear chronology and the dramatization of events, cannot fully capture the complexity of historical processes or the deeper causes behind historical changes, nor can they affirm historiography as a “true” science [ 15]. But the Annales historians themselves often reduce the historical narrative to a tradition of historiography. That is, they largely identify the narrative with a simple event-based political history which, although positivist and claiming to be scientific, was mostly written under the influence of national ideologies. When not reduced to this level of historiographical tradition, the narrative is viewed by the Annales school as merely a style of writing, an atavism, like something long ago borrowed from mythology, and now inadequate when history evolved into science. In opposition to that, Hayden White, in his work, approaches narrative as a key element in the construction of historical knowledge. According to White, the narrative is not just a neutral or transparent medium for conveying *facts* about the past; instead, it is a means through which history is interpreted, organized, and presented. Narrative, in White’s understanding, involves processes of selection, emphasis, and connection of historical events in ways that produce meaning and coherence. The narrative is fundamentally an interpretive act that makes the past understandable through contemporary categories, and by which the historian participates in the creation of the meaning of history, making historical writing a subjective and creative act.

Therefore, Annales historians and Hayden White approach the problem of historical narrative on two different levels. For the Annales School, narrative is primarily a style of presenting knowledge about history which, at least when dealing with shorter periods, can be viewed as a narrative but also as an almost static *tableau.* For White, however, narrative is the fundamental model for understanding history and organizing knowledge about it. Thus, while Annales historians believe that narrative must be abandoned in favour of a more scientific and structural mode of historical writing, White’s position is more nuanced and operates on two distinct levels. At the epistemological level, the level at which the historian first constitutes the historical field as an object of mental perception, White argues in *Metahistory* that narrativity is unavoidable. The act of prefiguring the historical field is, for White, a poetic and proto-narrative act that precedes any conscious methodological choice. That is, the historian must first construct a linguistic protocol by which the field and its elements become distinguishable and relatable, and this protocol is always already cast in a dominant tropological mode. In this sense, White cannot recommend the complete abandonment of narrative, because narrative structuring is not a stylistic choice but an epistemological precondition of historical knowledge as such. However, this does not mean that White is indifferent to the narrative form that the final historical text assumes. At the level of textual form and representation, White’s later work, particularly his essays from the 1980s and 1990s, repeatedly calls for experimentation with anti-narrative modes of historical writing, variously described as figural realism, writing in the middle voice, and anti-narrative non-stories. What White criticises at this level is not narrativity as such, but the specific form of narrative closure that imposes a moral and ideological resolution on historical events. The distinction between these two levels, narrativity as an epistemological condition, which is the main concern of this paper, and narrative as a form, is crucial for understanding both White’s critique of conventional historiography and the precise nature of his difference from the Annales School. The Annales historians sought to eliminate narrative at the level of textual form, without reflecting on the degree to which their own historical writing remained narratively prefigured at the deeper, epistemological level that White identifies. White, by contrast, accepts this deeper narrativity as inevitable while demanding a critical awareness of the consequences of moralising forms of conventional historical narrative. The ambivalence in White’s position on narrative is perhaps most clearly visible in *The Value of Narrativity in the Representation of Reality* ( 27), where White poses the rhetorical question of whether historians could produce historical knowledge without narrative closure that inevitably moralises the past. White asks whether it would be possible to narrativise history without moralising and his answer is implicitly affirmative, it is possible, and it is something historians should strive for. This is a crucial point that distinguishes White’s critique from a straightforward determinism about narrative. While White identifies the ideological function of narrative closure as a structural tendency in historiographical writing, he does not treat it as an insurmountable constraint. Instead, he positions it as a challenge, an invitation to experiment with forms of historical writing that resist the pull toward moral and teleological resolution. This dimension of White’s thought aligns him, somewhat paradoxically, with the anti-narrative ambitions of the Annales School, even as his theoretical framework for understanding narrative differs fundamentally from theirs.

Although these approaches of White and the Annales School differ and conflict greatly, one aspect of a common critique of narrative in historiography can nonetheless be discerned. That the narrative is not truly representational or even referential with respect to the past it deals with. It does not and cannot correspond to the past as it happened. This understanding of narrative leads to a critical reassessment of the referentiality of narrative historiography where each historiographical text not only relates to the past but also actively shapes it through the narrative and analytical choices of the historian. This problem of referentiality opens up questions about the scope of historiography, the possibility of it representing the real past, or conveying the truth about the past. As mentioned, for the Annales and like-minded historians, the path to solving this problem lies in rejecting the classical narrative, applying scientific methods, but also in attempting to write a total history of society by dealing with previously neglected topics. That is, for them, historiographical discourse differs from literature in two ways: first, because the subject matter it deals with is reality, not fiction, and second, because it applies the methodology of science. White’s approach is nowadays more interesting. He postulates the idea that there is no such essential difference between historiography and literature. The historian uses models and means of literature to convert events into a comprehensible whole, but even before that, he configures his own understanding of the past as a discursive field in which the subject matter of reality appears as fictional structures and figures. Or, as he explicitly writes in his 1978 article:

By the very constitution of a set of events in such a way as to make a comprehensible story out of them, the historian charges those events with the symbolic significance of a comprehensible plot-structure. Historians may not like to think of their works as translations of ‘fact’ into ‘fiction’, but this is one of the effects of their works. ( 28 on page 91–92)

Therefore, he uses the concept of *truth-claiming intention* to differentiate history from literature. As Nancy Partner summarises, for White “history is the kind of complex prose that encrypts truth-claiming intention and reaches for meaning by deploying the formal resources of language to create intelligible and logically connected emplotments of events.” ( 31 on page 169) However, in this context, the concept of *truth-claiming intention* is inadequate, or at least problematic. The assertion of a text’s truthfulness, its representational or even referential nature in relation to presumed historical reality is by no means exclusive to the domain of history as opposed to literature. The most obvious example of this is certainly realist literature, which Fredric Jameson defines as “a hybrid concept, in which an epistemological claim (for knowledge or truth) masquerades as an aesthetic ideal.” ( 32 on pages 3–7) This epistemological claim to truth in realism, or in other literary movements, is fundamentally no different from that in history [ 16]. Of course, while it is essential for a historical text to contain an assertion that it is truthful and referential to an actual event and period, literature may claim to be *truer than truth* because it does not need a real event to be referential to a given period, place, or other phenomenon. For describing non-narrative historiography, like that of the Annales historians, we might employ a slightly modified version of Jameson’s definition of realism [ 17]. Non-narrative historiography is a hybrid model in which the aesthetic ideal masquerades as an epistemological claim and method. The inability to fully replace literary character with the concept of photographic representation defines the hybridity of this model, which ultimately aims to substitutes aesthetics with epistemology. However, just as writing without an aesthetic ideal merely means writing with a different aesthetic ideal, so too does the attempt to create a history without narrative, even when this history is (almost) static, result only in the creation of a different narrative or, at the very least, leaves a larger role to the reader in forming their own narrative. After all, it is nearly impossible to read some of the great historians of the Annales School without noticing their exceptional literary skill and developed aesthetic. Perhaps the best example is *The Mediterranean*, in which Braudel, avoiding historical narrative, writes an extraordinary epic in which the Mediterranean itself emerges as the subject.

If we choose to follow White’s claim that the truthfulness of a historical narrative can be determined only in relation to chronology, or to the particular, we lose any possibility of using an external and objective measure to determine whether history is more truthful than literature, regardless of literature’s self-acknowledged fictional nature. Such an understanding of representation and truth departs from the positivist and empiricist ideas of the Annales School and the majority of mainstream historiography, designating the value of truth only to the realm of metaphor and allegory, a realm where literature can also rightfully claim its truthfulness. Given this, the approach to historical narrative is not a point at which historiography can draw a clear line of distinction from literature and prove its belonging to the firm sciences, not even in the case of the Annales School’s history without narrative. However, the conclusion of White’s general approach to history is not necessary to deny the potential of history to speak truth about the past. On the contrary, his critique opens new space for historiography but also calls for its redefinition, the abandonment of traditional notions of truth, and a shift in the field within which historiography functions [ 18].

By distinguishing between truth and its establishment, as well as between history and literature, Chris Lorenz emphasizes that the “intersubjective character of the rules of ‘doing history’ in contrast to ‘doing literature’” is what “constitutes history’s distinguishing hallmark as an empirical discipline.” ( 33 on page 326) [ 19] For him, the intersubjective character of the rules of history is essentially the process of exposition, argumentation, and questioning, namely, a process of validation according to various procedures and criteria applied by historians, including the assumption that the historian has first conducted research ( 33 on pages 326–327). However, Lorenz’s distinction is not entirely adequate. What he terms “doing literature” in no way implies that a writer does not undertake serious and rigorous research on his subject, and such research may align with the methods of history. Moreover, it is impossible to deny the intersubjectivity of literature, especially in certain genres. The most obvious example is likely the historical novel, in which public debate about historical accuracy, including chronological and factual accuracy, is entirely unavoidable. Nevertheless, the intersubjectivity of history, not only as a feature or characteristic but as the very definition and site of history-making, remains crucial. In this context, I refer to Foucault’s concept of a *regimes of truth.* In the interview *Truth and Power*, first published in 1977, Foucault argues that every society possesses “its regime of truth, its ‘general politics’ of truth; that is, the types of discourse which it accepts and makes function as true.” ( 35 on page 131) What Lorenz describes as the process of validation in history according to intersubjective rules, that is, the procedures and criteria of historiography, is elevated in Foucault’s framework to a broader social and historical context. This includes the “mechanisms and instances which enable one to distinguish true and false statements, the means by which each is sanctioned; the techniques and procedures accorded value in the acquisition of the truth; the status of those who are charged with saying what counts as true.” ( 35 on page 131).

Following Foucault’s concept of the regimes of truth, historiography should be defined through the relationship between the text and the society in which the question of truth is privileged as the dominant factor determining the nature of that relationship. Different societies, or different discourses, apply their own mechanisms, or power relations, to determine which statements, or, on a higher-level which narratives, are true and which are false. Alternatively, as Jouni-Matti Kuukkanen suggests in his shift from the framework of truth-functionality “to the framework of rational warrants and rational appropriateness,” the mechanisms define which statements and thesis are appropriate, fitting or unfitting, useful or useless, and so on. ( 36 on page 234) [ 20] By defining the relationship between the text and society (its audience) as the locus where historiography is constituted, both historiography and the text itself gain epistemic authority only in a retroactive relationship. That is, only when all the elements that traditionally constitute historiography, such as research and methodology, are recognized as science and as historiography that, to some extent at least, corresponds with the past. In this context, the key roles are not played by the source material, the methods used in the research itself, or even the modes of narrativization, but rather by the mechanisms of a given regime of truth. However, the assumption that certain source materials were used, that the research was conducted according to certain methodologically, and that the process of narrativization occurred are prerequisites for a text to be recognised as historiography, at least within the context of modern Western history. Only here is it possible to establish a distinction between history and literature that aligns with the narrativist critique of history. This distinction no longer stems from the intrinsic character or rules of historiography itself, such as the Annales School’s attempt to reject narrative, but is constituted as a specific construct. Historiography is accepted as a discourse with the exclusive capacity to speak the truth about history, based on two separations, the separation of the present from the past and the separation of the historian from history.

Defined in this way, as a discourse fixed through the relationship between text and society and with regard to regimes of truth, historiography can break free from certain self-imposed epistemic and methodological limitations. Following White’s conclusion about the political potential of the meaninglessness of history, we can infer that the meaninglessness of historiography can goad historians to construct histories with a meaning for which they alone are fully responsible. ( 34 on page 72) That is, to construct truly new histories and new historiographies. Or the evocate Marx oppening words of his classic *The Eighteenth Brumaire of Louis Bonaparte*, “The tradiotion of all past generations weighs like a nightmare on the brains of the living.” Furthermore, such a definition actually re-establishes the relevance of the theories and practices of the Annales School, including its attempt to write history without narrative. Moreover, in the context of modern Western historiography and contemporary Western society, which, at least as much as in the mid-twentieth century, grants a privileged position to the firm sciences as being more capable of speaking truth than the humanities, the Annales School can occupy a privileged position precisely because it masquerades as a firm science. In this way, the consequence of White’s critique of historical narrative and history in general, as well as the critiques of other theorists with of simmilar orientation, is not necessarily the disintegration of history as a discipline but its liberation from self-disciplining. In such a historiography, both the non-narrative narrative of history promoted by the Annales School and White’s tropological analysis could potentially be equally legitimate approaches to history.

## Ethics and consent

Ethical approval and consent were not required.

## Data availability

No data associated with this article.

## Footnotes

^1^
Journal was founded under the name “Annales d’histoire économique et sociale” in 1929, but it changed names often. From 1946 it was published as “Annales. Economies, sociétés, civilisations”, until 1994 when it was renamed “Annales. Histoire, Sciences Sociales”.

^2^
Hayden White’s observation from the late 1970s, that history since the mid-nineteenth century, has increasingly become “the refuge of all of those ‘sane’ men who excel at finding the simple in the complex and the familiar in the strange”, remains nearly as applicable today. Even if the trend White mentions no longer exists or has even been reversed, this positivist tendency is still present among historians and is evident in their search for inspiration in the historians of the Annales School. (Cf. 2 on page 50).

^3^
Here, I define historical narrative simply as a model for organizing diverse historical material into a chronological, or other, sequence to create a coherent yet potentially complex story, or narrative. Moreover, for a narrative to be historiographical, it must follow at least the general principles of the historiographical method and contain a truth-claiming intention.

^4^
The Annales School is typically divided into four successive generations that share common epistemological and methodological foundations, yet each develops its own unifying orientation. However, Lynn Hunt ( 7 on page 213) points out that from the third generation onward, specifically after Braudel ceased to edit the journal and retired from his chair at the Sixth Section of the École Pratique des Hautes Études, a disintegration of the Annales paradigm can be observed, with a growing fragmentation of common research orientations and theoretical principles. The results of this disintegration are evident several decades after the end of the third generation of the Annales School. For example, Ivan Jablonka places himself within the Annales historian tradition. However, his seminal work *History is a Contemporary Literature* ^6^, published in 2014, theoretically breaks entirely with the Annales School tradition regarding the relationship between history and literature as it was embodied up to the third generation. If we set the relationship between history and literature as one of the fundamental questions of analysis, when it comes to Annales historians, including those after the third generation, we truly cannot speak of them as a school. Instead, we can perhaps accept the claim of some *Annales* historians that they were connected by a similar spirit of openness to new methods, research orientations, and disciplines. However, such a broadly defined shared characteristic of the Annales historians would mean that they were connected by a similarity in their potential to differ, not only from other contemporary historiographical orientations but also from each other, which would in turn make it impossible to describe them as a group with at least minimal coherency.

^5^
Speaking about the reception of his works, Stephen Bann notes that White found himself under a “general embargo” in France, citing Roland Barthes as an example ( 9 on pages 145–146). Barthes’ ideas were frequently used by White, yet Barthes himself never referenced White and may not have even read him. Philippe Carrard adds that “White’s name is similarly absent from the works of several French scholars interested in the philosophy of history that White discusses on various occasions and at different lengths, such as Michel Foucault, Paul Veyne, and Michel de Certeau.” ( 10 on pages 1–6) However, Carrard believes that “general embargo” is too strong a term and points out that Paul Ricoeur devoted some attention to White in *Temps et récit* in the mid-1980s. He also highlights that professional historians were even slower than Ricoeur in engaging with White’s work, with the turning point coming only in 1992, when White, at the invitation of Roger Chartier, spent six weeks at the École des Hautes Études en Sciences Sociales, delivering several lectures.

^6^
François Furet argued that the *Annales* were “more than a journal – and less than a doctrine,” but that the perception of *Annales* historians as a group sharing a unified concept of the discipline, or as a school, is mistaken and stems from institutional logic and attempts to explain the decades-long rivalry between the Sixth Section of the École Pratique des Hautes Études, where the *Annales* historians were based, and the faculties of letters at universities, particularly the Sorbonne ( 17 on pages 390–391). On the other hand, although Furet does not use the concept of the Annales School, he speaks of generations of historians developing under the intellectual guardianship of earlier *Annales* historians, which actually implies the existence of some form of coherent intellectual tradition and school. Cf. ( 18 on page 51).

^7^
The departure from the theory of historiography is evident even in the very title of Bloch’s book. Its original French title is *Apologie pour l’histoire ou Métier d’historien.* This is generally translated into English as *The Historian’s Craft*, into Croatian as *Apologija historije ili zanat povjesničara*, and similarly into other languages. The term *métier*, *craft*, or *zanat* emphasizes the technical or practical aspect, implying that the nature of the historian’s work is akin to that of a craftsman who applies his skills, techniques, and specific tools without imprinting much of his creative thought on the produced work. In other words, for Marc Bloch the historian is a craftsman, not an artisan or an artist.

^8^
Traditionally understood history, shaped as a narrative through a set of causal events within a certain time period, is determined by a teleological principle. Events and phenomena preceding something in time explain what follows them, but it is only what follows that gives meaning to these events and phenomena, constituting them as history and as a subject of historiographical research.

^9^
Hans Kellner, in his essay “Disorderly Conduct: Braudel’s Mediterranean Satire” ( 19), offers a reading of The Mediterranean that cuts across the grain of the Annales School’s own self-understanding. Rather than treating Braudel’s work as a successful escape from narrative, Kellner argues that it can be read as a form of satirical emplotment in White’s sense, a text that, by systematically inverting the conventions of event-based narrative history, ironically reproduces many of narrative’s structuring functions. This complicates any straightforward opposition between the Annales approach and White’s narrativism, and suggests that the Annales School’s rejection of narrative may itself be a narrative strategy of a particular kind.

^10^
Braudel compares the historical *tableau* to today’s sociological photography, noting that it is no “more truthful” than its historiographical counterpart. However, it should be noted that there is a departure from the idea of photography or a critique of the concept of sociological photography of society. Unlike photographic replication, where the author’s influence is mostly limited to the choice of what is photographed, the *tableau* implies a faithful representation created through the active work of the artist, or here, the historian-craftsman (cf. 22 on page 8).

^11^
Among the Annales historians, the one who advanced furthest in this direction was Le Roy Ladurie, who, in his series of essays on historiography, even emphasized that “history that is not quantifiable cannot claim to be scientific.” ( 23 on page 15)

^12^
As Herman Paul has notes in his intellectual biography of White, White’s engagement with narrative underwent several shifts between the publication of Metahistory in 1973 and his later essays. Paul shows that, while White in the 1970s primarily analysed narrative as a tropologically prefigured form imposed on historical material, his later work increasingly stressed the ideological content that narrative form itself carries, what White called the content of the form. ( 6) However his shift in focus does not negate basic theoretical assumption of the necessity of narrative prefiguration put forth in *Metahistory.*

^13^
There is no history without that element of the *foreign.* Every presumed real event, from the moment it is recorded or documented, is already selected and defined by the chronicler or author and later disciplined by the historian. Thus, the chroniclers of the Saint Gall monastery record only those events they deem relevant to the narrative in accordance with which they write, or into which they incorporate events. Additionally, an important aspect that White does not address here is the question of how historiography is read and utilized.

^14^
White’s thesis in this way undermines the main foundation of history since its separation from myth, its ability to speak the truth about the past. Such ideas were especially problematic in the context of the *Historikerstreit*, which took place in Germany from the latter half of the 1980s but also significantly influenced other European historiographies. Consequently, a frequent and ultimate critique of White raises the issue of the Nazi death camps, questioning whether White’s approach implies a denial or relativization of the Holocaust.

^15^
In this sense, the historiography of the Annales School and their approach to historical narrative can be seen as a continuation and elaboration of the traditional realist conception. In other words, it is a continuation of the Rankean idea that history should be written *wie es eigentlich gewesen*, in a context where past reality is not considered to be narratively determined.

^16^
White himself states that the creative process is fundamentally the same for both the historian and the novelist or poet, as both attempt to create an explanation “by endowing what originally appears to be problematical and mysterious with the aspect of a recognizable, because it is familiar, form.” Additionally, White emphasizes that his literary analysis of history writing raises the broader question of the truthfulness of literature itself. Cf. ( 28 on page 98), ( 29 on pages 27–28).

^17^
Here, it is worth returning to Furet’s earlier assertion about narrative in historiography as something that, in the name of art and sensitivity, renders the historian’s mind and intelligence secondary. ( 17 on page 406)

^18^
White, in his general critique of history and historiography, saw immense political potential in liberating individuals and societies from the constraints imposed by history. In his essay *“*The Politics of Historical Interpretation *,”* he argues that modern ideologies, much like eschatological religious myths, “deprive history of the kind of meaninglessness that alone can goad living human beings to make their lives different for themselves and their children, which is to say, to endow their lives with a meaning for which they alone are fully responsible.” White concludes that the function of historical narrative is disciplinary, anti-utopian, and grounded in the assumption that history is “a comprehensible process the various parts, stages, epochs, and even individual events of which are transparent to a consciousness endowed with the means to make sense of it.” ( 34 on pages 72–73)

^19^
Similar theses are presented by Jouni-Matti Kuukkanen, who, under the influence of the “narrativist insight,” accepts the abandonment of the concept of historical truth based on the theory of correspondence, and consequently the truth-functionality of history, but not the rationalist foundations of disciplinary historiography. Kuukkanen, in fact, builds upon the idea presented by Lorenz by attributing to historiography the character of a performative practice. For him, historians, through various modes of reasoning and argumentation, “put forward arguments in the form of the books they write and try to bring about a change in their argumentative context.” ( 35 on page 227)

^20^
Kuukkanen seeks to address the problem of truth and the inadequacy of truth-function, as highlighted by narrativists, by situating the mechanisms of historiographical proof and refutation within a “framework of rational warrants and rational appropriateness.” He emphasizes that historians and historiography operate in a specific argumentative context in which they “have to make their moves in the game of argumentation shaped by decades of discourse, taking the existing views into account; they must be ready to defend their claims with regard to the prevailing state of knowledge.” Kuukkanen argues that epistemic authority does not stem solely from source material or methodology but is retroactively established through a successful intervention in the argumentative context or the defense of a thesis, with the rules of the context being spatially and politically determined. However, he points out that the rules of argumentation are always based on rational grounds, stating that “historiographical discourse takes place in a politicized context, but on rational grounds.” The problem with Kuukkanen’s thesis is that, contrary to his claim, it leaves a privileged place for truth since the framework of rational warrants and appropriateness can only be established in relation to a presumed but unattainable value of truth, which determines what is warranted and appropriate. Another issue is the lack of critique regarding what determines “rational grounds,” specifically how the concept of rationality is itself entirely politically determined. As a result, Kuukkanen’s theses function only partially within the type of historiographical field dominated by mainstream historians but fail to operate in the broader social context, where the value of historiography is shaped by relationships of differing natures and different types of rational grounds. ( 36 on pages 240–242).
