# Supplementary material for: The Question of the Narrative in Historiography: Between the Annales School and Hayden White
Source: Open Res Eur. 2026 Apr 2;5:259. Originally published 2025 Aug 27. [Version 2] doi: 10.12688/openreseurope.20777.2 (PMC13189153; doi:10.12688/openreseurope.20777.2)
Supplement: Peer review history

# Reviewer report: Reviewer response for version 2

**DOI:** 10.21956/openreseurope.25271.r71872

**Published:** 2026-05-06

**Authors:** Christina Banalopoulou

**Affiliations:** 1 University of Milan, Milan, Lombardy, Italy

This article generates a productive dialogue between the Annales school’s understanding of narrative and that of Hayden White, focusing on how each conceives of the role it plays in historical discourse. As the author notes, this comparative investigation is an underexplored area of inquiry, and the article makes a genuine contribution by rendering the internal contradictions of each approach analytically explicit and then tracing common ground between them.

 The article’s strongest aspect is its articulation of the contradictions of these traditions, which lays the ground for their conceptual intersections as well. The tensions between the Annales’ rejection of linear causality and their shift in focus from the institutional to the everyday as a unit of historiographical analysis, on the one hand, and their rejection of the narrative as an obstacle to epistemological scientificity, on the other, are thoroughly unpacked. Hayden White’s position is examined with similar thoroughness. For White, historical discourse is always-already *figural* and narrativized. Therefore, the question is not whether historical writing is narrated (it inevitably is) but what kinds of literary engagement allow us to understand the constitutive, presentational, and productive — rather than merely representational — dimensions of history. The article then productively identifies a shared critical ground. Both the Annales and White, despite their differences, challenge the narrative’s representational claims.

**Points for Revision**

 Content: The author situates the dialogue around questions of truth and referentiality, yet does not fully address why revisiting this theoretical gap is important now. The article would benefit from a more explicit articulation of the stakes of this theoretical inquiry. Given that the article engages successfully with questions of narrative, truth, and the epistemological status of historical discourse, it would be beneficial to connect this inquiry to contemporary debates. Even a brief framing of why this dialogue is timely would significantly strengthen the article’s intervention.

 Style and Literature Engagement: I would invite the author to avoid strong claims that are not directly supported by evidence. The opening, for instance, risks implying hierarchies of historiographical traditions and marginalizing others. I would also encourage the author to include citations when listing scholars and their works. The author is also encouraged to engage more widely with existing scholarship. For instance, Saidiya Hartman’s “critical fabulation” as articulated in “Venus in Two Acts,” *small axe* 26 (June 2008): 1-14, could shed light on the stakes of the relations between storytelling, narrative, and history. Similarly, Sylvia Wynter’s “Unsettling the Coloniality of Being/Power/Truth/Freedom: Towards the Human, After the Man, Its Overrepresentation – An Argument,” *CR:* *The New Centennial Review* 3(3) (2003): 257-337 in terms of the discussion on truth and narrative. Finally, Walter Mignolo and María Lugones’s work and frameworks, more broadly, could contribute to the essay’s critical toolbox of interrogating how historical narratives are constructed, to whose benefit, and to what ends.

 Organization: Because of its theoretical density, dividing the article into sections could make its contribution even clearer.  

 Overall, this is a carefully argued and theoretically rich essay that makes an important contribution to discussions around narrative, truth, and historical discourse. The revisions suggested above are relatively minor and aim primarily to sharpen the essay’s articulation of its own stakes, particularly its relevance to contemporary debates around truth and representation in which the questions it raises are urgently implicated.

If any, are all the source data and materials underlying the results available?

No source data required

Is the work original in terms of material and argument?

Yes

Is the work clearly and cogently presented?

Yes

Is the argument persuasive and supported by evidence?

Yes

Does the research article contribute to the cultural, historical, social understanding of the field?

Yes

Does it sufficiently engage with relevant methodologies and secondary literature on the topic?

Partly

Reviewer Expertise:

Theatre and Performance Studies, Performative Turn in History/Historiography, Decolonial Theory

I confirm that I have read this submission and believe that I have an appropriate level of expertise to confirm that it is of an acceptable scientific standard.

# Reviewer report: Reviewer response for version 2

**DOI:** 10.21956/openreseurope.25271.r71874

**Published:** 2026-04-24

**Authors:** Alina Bako

**Affiliations:** 1 Lucian Blaga University of Sibiu, Sibiu, Romania

The article written by Karlo Držaić addresses a relevant topic that is well grounded in contemporary debates within the theory of historiography, focusing on the relationship between narrativity and the construction of historical knowledge through a comparison between the Annales School and the thought of Hayden White. The approach is coherent and well structured, and the author demonstrates a solid command of the relevant scholarly literature.

 One of the main strengths of the article lies in the clarity and coherence of its argumentation. The author succeeds in organizing a complex theoretical problem into an accessible discourse without excessively simplifying the concepts involved. The comparison between the Annales School and White is well developed and pertinent, particularly in its emphasis on the epistemological differences concerning the status of narrative. Moreover, the article is well documented, drawing on relevant and up-to-date references, which lends it academic solidity and a strong positioning within the theoretical field.

 It is important to note that the text does not aim to provide an innovative contribution in the strict sense, but rather offers a conceptual synthesis of two major traditions in historiography. In this regard, the objective is successfully achieved, and the article functions effectively as a tool for theoretical clarification and systematization.

 At the same time, a few minor adjustments could further strengthen the analysis. First, a more explicit clarification of Hayden White’s position—particularly the distinction between the epistemological level of narrativity and its textual manifestation—would enhance the precision of the argument. Second, although the article is primarily theoretical, the inclusion of a brief applied example would help illustrate the arguments more concretely and reinforce the demonstrative dimension of the discussion.

 Overall, the article is well written, coherent, and academically appropriate. Although it does not seek to advance a radically new theoretical contribution, its value lies in its ability to clearly synthesize and organize a complex debate. In its current form, the text is publishable and will be useful for readers interested in the theory of historiography. The English is correct and appropriate for academic purposes, even if it occasionally retains a slight rigidity characteristic of a more theoretical style.

If any, are all the source data and materials underlying the results available?

Yes

Is the work original in terms of material and argument?

Yes

Is the work clearly and cogently presented?

Yes

Is the argument persuasive and supported by evidence?

Yes

Does the research article contribute to the cultural, historical, social understanding of the field?

Yes

Does it sufficiently engage with relevant methodologies and secondary literature on the topic?

Yes

Reviewer Expertise:

World Literature

I confirm that I have read this submission and believe that I have an appropriate level of expertise to confirm that it is of an acceptable scientific standard.

# Reviewer report: Reviewer response for version 2

**DOI:** 10.21956/openreseurope.25271.r71871

**Published:** 2026-04-17

**Authors:** A. Arun Daves

**Affiliations:** 1 Jawahar Science College, Neyveli, Tamil Nadu, India

This article examines the role of narrative in historiography through a comparative analysis of the Annales School and Hayden White. It argues that while the Annales historians reject narrative to establish history as a science, White redefines narrative as an unavoidable epistemological condition shaping historical meaning. The paper successfully highlights both the tension and the underlying convergence between these positions, particularly in their shared skepticism toward narrative’s representational capacity.

 The work is original and demonstrates strong engagement with relevant theoretical frameworks and secondary literature. The argument is persuasive, well-supported, and contributes meaningfully to historiographical and philosophical debates. However, the presentation is at times dense, with lengthy paragraphs and complex sentences that may hinder clarity. The author should refine these sections for conciseness and improve the flow of argumentation.

 No empirical data issues arise, as the study is purely theoretical. To strengthen the article further, the author should:

1. Simplify and streamline dense passages.
2. Clarify the central argument in the introduction and conclusion.
3. Reduce minor repetition in theoretical explanations.

With these minor revisions, the article is scientifically sound and suitable for indexing.

If any, are all the source data and materials underlying the results available?

No source data required

Is the work original in terms of material and argument?

Yes

Is the work clearly and cogently presented?

Partly

Is the argument persuasive and supported by evidence?

Yes

Does the research article contribute to the cultural, historical, social understanding of the field?

Yes

Does it sufficiently engage with relevant methodologies and secondary literature on the topic?

Yes

Reviewer Expertise:

English Literature, Language and Linguistics, Indian English Literature, American Literature, Commonwealth Literature, Creative Writing

I confirm that I have read this submission and believe that I have an appropriate level of expertise to confirm that it is of an acceptable scientific standard.

# Reviewer report: Reviewer response for version 2

**DOI:** 10.21956/openreseurope.25271.r71826

**Published:** 2026-04-10

**Authors:** Jakub Muchowski

**Affiliations:** 1 Department of History, Uniwersytet Jagielloński, Kraków, MAŁOPOLSKIE, Poland

The article provides a comprehensive and original comparison of the approaches to the concept of narrative developed by historians from the Annales School and Hayden White. The author has made insightful and creative revisions to an earlier version of the paper.

If any, are all the source data and materials underlying the results available?

No source data required

Is the work original in terms of material and argument?

Partly

Is the work clearly and cogently presented?

Yes

Is the argument persuasive and supported by evidence?

Partly

Does the research article contribute to the cultural, historical, social understanding of the field?

Partly

Does it sufficiently engage with relevant methodologies and secondary literature on the topic?

Partly

Reviewer Expertise:

My research focuses on the theory of historical writing, Polish memory cultures, transnational history of Poland and labour history. My ongoing work centres on reading historical writing, transnational relations between Poland and South Africa and forms of invisibility of labour in industrial museums.

I confirm that I have read this submission and believe that I have an appropriate level of expertise to confirm that it is of an acceptable scientific standard.

# Reviewer report: Reviewer response for version 1

**DOI:** 10.21956/openreseurope.22480.r66561

**Published:** 2026-01-21

**Authors:** Jakub Muchowski

**Affiliations:** 1 Department of History, Uniwersytet Jagielloński, Kraków, MAŁOPOLSKIE, Poland

The author addresses the important question of how the Annales school and Hayden White differ in their views on the role of narrative in producing historical knowledge. In doing so, he shows that he has a thorough understanding of the relevant scholarship. However, he does not explain how his paper engages with current debates in historical theory. The paper lacks a reference to Hans Kellner's article 'Disorderly Conduct: Braudel's Mediterranean Satire', which is relevant to the task at hand as it relates Braudel's views to White's.  Furthermore, the article could refer to the detailed explanations of White's views on the question of historical narrative presented by Herman Paul and Kalle Pihlainen in their books.

 As I mentioned earlier, the author compares the Annales school's approach to historical narrative with that of Hayden White. As he acknowledges, this framing of the problem may give rise to two concerns. Firstly, Annales historians did not form a uniform community, and although their theoretical views were similar, they differed in some respects, including those concerning narrative. Secondly, this group devoted little attention to theory and only partially articulated their views on the role of narrative in historical writing. This means that one side of the proposed comparison is unclear. The author acknowledges this lack of clarity and addresses it in his discussion. It would be beneficial to supplement these passages concerning the position of the Annales with specific examples of Annales scholars' views on narrative. For instance, Jacques Le Goff's concept of anti-biography could be referenced.

 Hayden White’s views on historical narrative seem easier to grasp. However I wonder if these have been accurately characterised. White certainly emphasised the key role of narrative in the production of historical knowledge by historians. At the same time, however, he also criticised this mode of constructing history for various reasons, as the author notes. In the 1970s, he regarded narrative as a fundamental means of organising research material imposed on historians by language-like structures of thought (tropes). By the 1980s, he believed that narrative forms would smuggle ideology into historical knowledge; the content of the narrative form would be ideology. However, I disagree with the author's assertion that 'Annales historians believe that narrative must be abandoned, but White cannot even make such a suggestion because narrative is unavoidable'. While White did emphasise the difficulty of avoiding the narrative mode of configuring historical knowledge, he himself repeatedly suggested, that historians should experiment with anti-narrative forms of constructing historical knowledge, although he did not explain how. White repeatedly calls for a alternative kind of historical writing in his works. He refers to this as 'figural realism', 'writing in the middle voice', 'modernist realism' and 'anti-narrative non-stories'. The essential feature of this approach is anti-narrativity. In 'The Value of Narrativity in the Representation of Reality', mentioned by the author, White asks: 'Could we answer that question without providing a narrative account of the history of objectivity itself, one that would predetermine the outcome of the story we would tell in favour of the general moral? Could we ever narrativise without moralising?' White expects us to answer 'yes'; that it is possible to create historical knowledge without narrative closure that moralizes.

If any, are all the source data and materials underlying the results available?

No source data required

Is the work original in terms of material and argument?

Partly

Is the work clearly and cogently presented?

Yes

Is the argument persuasive and supported by evidence?

Partly

Does the research article contribute to the cultural, historical, social understanding of the field?

Partly

Does it sufficiently engage with relevant methodologies and secondary literature on the topic?

Partly

Reviewer Expertise:

My research focuses on the theory of historical writing, Polish memory cultures, transnational history of Poland and labour history. My ongoing work centres on reading historical writing, transnational relations between Poland and South Africa and forms of invisibility of labour in industrial museums.

I confirm that I have read this submission and believe that I have an appropriate level of expertise to confirm that it is of an acceptable scientific standard, however I have significant reservations, as outlined above.

# Reviewer report: Reviewer response for version 1

**DOI:** 10.21956/openreseurope.22480.r64137

**Published:** 2025-11-24

**Authors:** Arif Saefudin, Muhamad Arif

**Affiliations:** 1 sOCIAL SCIENCES, Universitas Islam Negeri Syarif Hidayatullah Jakarta, South Tangerang, Banten, Indonesia

1. The topic raised is an important part of the discourse on modern historiography theory. The author has presented a neat and systematic synthesis, but this article does not fully offer new conceptual contributions that expand on existing theories. To strengthen its originality, the author could develop a conceptual framework such as a model of historiographical intertextuality that connects narrative and social structure in historical practice.
2. In addition, the analysis would be more vivid if it were supplemented with case studies (e.g., readings of specific historical works) to show how the theories of Annales and White are applied or even debated in the practice of historical writing. It is also important to explicitly state the novelty of this article compared to previous literature, such as readings by Ankersmit, Jenkins, or Lorenz.
3. Although the literature used is broad and relevant, the methodological approach has not been explicitly explained. The author appears to use conceptual and hermeneutic analysis, but without describing the methods or criteria of analysis that form the basis for comparison between the two traditions. Therefore, a methodology subsection is needed to explain the analytical approach, such as conceptual comparison, discourse analysis, or intellectual genealogy, as well as the criteria for selecting and interpreting sources so that readers understand the limitations and validity of the analysis. The use of more recent secondary literature (post-2010) would also reveal the position of this article in the debate on narrativism and contemporary historiography.
4. The main argument is consistent, but textual support for the claims is still limited. Many quotations serve as illustrations rather than as argumentative evidence. To strengthen the claims, a textual analysis (close reading) of the main works of White and Braudel is needed, as well as a presentation of the counter-positions of historians who criticize White's views, such as Iggers or Evans, accompanied by the author's responses. Thus, quotations serve not only an informative purpose, but also an argumentative and reflective one.
5. As a conceptual and philosophical article, this study does not require empirical data. However, the author could add visual elements (such as conceptual diagrams or comparison charts) to clarify the relationships between key concepts. Although its contribution lies in philosophical reflections on how history is understood and written, this article would be stronger if it were linked to broader social and cultural dimensions, such as its influence on contemporary historical practices, the politics of memory, or the construction of collective identity.
6. Overall, this article has strong potential for publication after substantive revision, particularly in terms of methodology, deepening of textual evidence, and strengthening of its applied relevance to historiographical practice and the socio-cultural context.

If any, are all the source data and materials underlying the results available?

Partly

Is the work original in terms of material and argument?

Partly

Is the work clearly and cogently presented?

Partly

Is the argument persuasive and supported by evidence?

Partly

Does the research article contribute to the cultural, historical, social understanding of the field?

Partly

Does it sufficiently engage with relevant methodologies and secondary literature on the topic?

Partly

Reviewer Expertise:

history; education

We confirm that we have read this submission and believe that we have an appropriate level of expertise to confirm that it is of an acceptable scientific standard, however we have significant reservations, as outlined above.
